# Editorial: Beyond Cardiovascular Disease: Challenging New Pathways in Lipid and Lipoprotein Metabolism

**DOI:** 10.3389/fcell.2022.963463

**Published:** 2022-06-30

**Authors:** Marisa Passarelli, Shinji Yokoyama, Andrei Sposito

**Affiliations:** ^1^ Laboratório de Lípides (LIM 10) do Hospital das Clínicas (HCFMUSP) da Faculdade de Medicina da Universidade de São Paulo, São Paulo, Brazil; ^2^ Programa de Pós-Graduação em Medicina, Universidade Nove de Julho, São Paulo, Brazil; ^3^ Food and Nutritional Sciences, Chubu University, Kasugai, Japan; ^4^ Atherosclerosis and Vascular Biology Laboratory (Atherolab), Cardiology Division, University of Campinas (UNICAMP), Campinas, Brazil

**Keywords:** HDL, sepsis, immunity, lipoproteins, lipid metabolism

The co-incidence of xanthomatosis and angina pectoris was firstly reported by Carl Muller (1939) and opened a large room for several decades of investigations into the interconnection between cholesterol accumulation and cardiovascular disease (CVD) apud (Muller, 1987). Later, with the development of the analytic ultracentrifugation technique for lipoproteins isolation by John Gofman in 1949, the comprehension of the role of these cholesterol-carrier particles on CVD enormously increased (Gofman et al., 1949).

The results from the Framingham Study defined a contrasting role played by low-density lipoproteins (LDL) and high-density lipoproteins (HDL), respectively in the stimulation and prevention of atherosclerosis (Gordon et al., 1977; Andersson et al., 2019). In parallel, the Seven Countries Studies introduced clues on the dietary fat contribution to lipoprotein profile and CV risk (Keys, 1980). Recently, the detailing of the genetic source for dyslipidemias explored the contribution of monogenic and polygenic lipid traits in lipid disorders and favored the development of new drugs.

Although classically related to cardiovascular disease and atherosclerotic burden, nowadays plasma lipoproteins relate to the development and prognosis of other diseases. They included evidence regarding the association among lipoproteins with diabetes mellitus, many types of cancer, and inflammatory and infectious diseases.

HDL functionality and proteins related to its metabolization in the plasma and lymphatic compartment encompass a myriad of pathophysiological processes. The advances in technical approaches to the evaluation of the HDL subfractions based on 2-D electrophoresis, ultracentrifugation, immunological methods, and magnetic nuclear resonance allowed the comprehension of this heterogeneous lipoprotein family, its generation, maturation, and actions in several diseases. The HDL proteome and lipidomics reveal a large range of peptides and bioactive lipids that define HDL functionality. It includes the HDL’s ability in removing excess cholesterol from peripheral cells, the so-called reverse cholesterol transport, but also its actions as an antioxidant, anti-inflammatory, hematopoietic, and in improving endothelial-mediated vasodilation, plaque stability, and glucose homeostasis. Moreover, the role of HDL as a cargo particle for microRNA and its delivery to target cells *via* SR-B1 also enrolls this lipoprotein in the modulation of cell function in different health conditions.

Importantly, all HDL activities that seem to modulate CV risk and the pathophysiology of many diseases are not strictly conditioned to the traditional laboratory metrics of the HDL content in cholesterol or apolipoprotein (apo) A-I plasma levels. This became crystal clear after many negative clinical trials that tested the protective effect of enhancing plasma HDL-cholesterol levels (Barter et al., 2007; Schwartz et al., 2012; Group et al., 2017; Lincoff et al., 2017) and also after observational studies with Mendelian randomization which failed in demonstrating the association between increased HDL-cholesterol or apo A-I and CVD outcomes (Frikke-Schmidt et al., 2008; Johannsen et al., 2009; Haase et al., 2010; Voight et al., 2012).

Acute-phase response proteins that are markedly elevated during inflammation and infection constitute a large family of proteins associated with the HDL particle. HDLs are the major lipoproteins that bind and neutralize lipopolysaccharide and lipoteichoic acid, components of the outer membrane of respectively, Gram-negative and Gram-positive bacteria (Meilhac et al., 2020). These interactions are mediated by cholesteryl ester transfer protein (CETP), phospholipid transfer protein, and lipopolysaccharide-binding protein associated with the HDL particle.

A large number of studies reported a negative association between HDL-cholesterol levels and mortality or adverse clinical outcomes in sepsis and the beneficial effect of the infusion of HDL mimetics or reconstituted HDL on morbimortality in animal models of sepsis (Morin et al., 2015). However, controversies still exist and there are several reports of a negative or absent association between changes in plasma HDL-cholesterol and the sequential assessment score of organ failure (SOFA) or mortality in sepsis. Further investigations have sought to identify components in the HDL lipidome and proteome that may act as modulators of the sepsis response or as prognostic markers. In this Research Topic, Reisinger et al. present evidence on the role played by the lecithin cholesterol acyltransferase (LCAT) activity in discriminating intensive care unit septic patients from non-septic subjects and even predicting mortality outcomes in sepsis. LCAT activity was a better predictor of mortality in sepsis as compared with serum amyloid A that besides being an inflammatory marker was not associated with mortality rate.

HDL also plays a role in parasitic infections and seems to contribute to innate immunity (Grao-Cruces et al., 2022). On the other hand, HDL receptors, such as SR-B1 and ABCA-1 favor virus entry into cells, and their antagonism is proposed as a therapeutically approach against virus infection. The cholesteryl ester transfer protein (CETP) dictates HDL-cholesterol levels in plasma by exchanging esterified cholesterol and triglycerides between HDL and apo B-containing lipoproteins. Then, CETP inhibitors are potential contributors to raising HDL-cholesterol, although controversies persist considering its role in neutralizing bacterial endotoxins favoring the HDL’s anti-inflammatory properties, and failure in CVD outcomes. In this Research Topic, Yokoyama presents a review of the role of HDL in providing lipids for *Schistosoma japonicum* egg development *via* a CD-36-like protein expressed in the parasite. The Japanese deficiency of CETP by enlarging HDL particles makes it a poor substrate for parasite maturation and the prevalence of CETP mutation in East Asia overlaps with endemic areas of *S. japonicum* infection possibly representing an adaptive mechanism that allows for preventing major and fatal complications of schistosomiasis.

Fatty acids transported into cells modulates lipid and glucose homeostasis, energy balance, cell cycle, neurological development, tumorigenesis, and immunological system. In this Research Topic, Xu et al. presented a detailed review of the biological regulation and function of fatty acid-binding protein 5 (FABP5) as a fatty acid transporter but also as an intracellular signaling molecule. Pieces of evidence are brought about the association of FABP5 levels with metabolic diseases and its role as an independent risk factor for CVD. In part, these relate to the role of FABP5 in modulating inflammation and abrogating macrophage cholesterol efflux mediated by HDL but also to a plethora of actions on lipid homeostasis, metabolic disorders, and other diseases.

Adaptive thermogenesis impacts plasma lipid and lipoprotein profiles. Evangelakos et al. evidenced a role for bile acids in modulating brown adipose tissue thermogenesis that ultimately relates to the impairment in the lipoprotein lipase-mediated hydrolysis of triglyceride-rich lipoprotein in Cyp7b1 knockout mice. Further studies should address the impact of bile acids on plasma lipoprotein profile and functionality.

In summary, detailing the players and pathways in lipid metabolism, particularly on HDL, has made a great contribution to the prevention of CVD. Further studies will certainly guide us through new therapeutic targets for mitigating the still elevated residual risk of atherosclerotic CVD as well as for a range of other acute and chronic diseases.
